# The Fatty Acid and Protein Profiles of Circulating CD81-Positive Small Extracellular Vesicles Are Associated with Disease Stage in Melanoma Patients

**DOI:** 10.3390/cancers13164157

**Published:** 2021-08-18

**Authors:** Giovanni Paolino, Veronica Huber, Serena Camerini, Marialuisa Casella, Alberto Macone, Lucia Bertuccini, Francesca Iosi, Elisa Moliterni, Serena Cecchetti, Irene Ruspantini, Flavia Chiarotti, Elisabetta Vergani, Luca Lalli, Carla Raggi, Antonella Di Biase, Stefano Calvieri, Santo Raffaele Mercuri, Luana Lugini, Cristina Federici

**Affiliations:** 1 Department of Dermatology, Sapienza University of Rome, 00185 Rome, Italy; paolino.giovanni@hsr.it (G.P.); elisa.moliterni@uniroma1.it (E.M.); stefano.calvieri@uniroma1.it (S.C.); 2 Unit of Dermatology and Cosmetology, IRCCS University Vita-Salute San Raffaele, 20132 Milan, Italy; mercuri.santoraffaele@hsr.it; 3 Unit of Immunotherapy of Human Tumors, Fondazione IRCCS Istituto Nazionale dei Tumori, 20133 Milan, Italy; veronica.huber@istitutotumori.mi.it (V.H.); elisabetta.vergani@istitutotumori.mi.it (E.V.); luca.lalli@istitutotumori.mi.it (L.L.); 4 Core Facilities Mass Spec Unit, National Institute of Health, 00161 Rome, Italy; serena.camerini@iss.it (S.C.); marialuisa.casella@iss.it (M.C.); irene.ruspantini@iss.it (I.R.); 5 Department of Biochemical Sciences “A. Rossi Fanelli”, Faculty of Pharmacy and Medicine, Sapienza University of Rome, 00185 Rome, Italy; alberto.macone@uniroma1.it; 6 Core Facilities Electron Microscopy Unit, National Institute of Health, 00161 Rome, Italy; lucia.bertuccini@iss.it (L.B.); francesca.iosi@iss.it (F.I.); 7 Core Facilities Confocal Microscopy Unit, National Institute of Health, 00161 Rome, Italy; serena.cecchetti@iss.it; 8 Reference Center for Behavioral Sciences and Mental Health, National Institute of Health, 00161 Rome, Italy; flavia.chiarotti@iss.it; 9 National Centre for the Control and Evaluation of Medicines, National Institute of Health, 00161 Rome, Italy; carla.raggi@iss.it; 10 Department of Food Safety, Nutrition and Veterinary Public Health, National Institute of Health, 00161 Rome, Italy; antonella.dibiase@iss.it; 11 Department of Oncology and Molecular Medicine, National Institute of Health, 00161 Rome, Italy

**Keywords:** melanoma patients, plasma small extracellular vesicles, proteomics, fatty acids, C18:0/C18:1 ratio

## Abstract

**Simple Summary:**

Early detection of cutaneous melanoma is the key to increasing survival and proper therapeutic adjustment, especially in stages II–IV. We investigated whether the fatty acid (FA) and protein compositions of small extracellular vesicles (sEV) expressing CD81, derived from the plasma of stage 0–I, II and III–IV melanoma patients, could reflect disease stage. Results showed a higher content of FA and differences in C18:0/C18:1 ratio, a marker of cell membrane fluidity, that distinguished patients’ CD81sEV from those of healthy donors (HD). By proteomic analysis (identifier PXD024434) we identified significant increases in CD14, PON1, PON3 and APOA5 in stage II CD81sEV compared to HD. In stage III–IV, CD81sEV’ RAP1B expression was decreased. These stage-related signatures may support the potential of sEV to provide information for early diagnosis, prediction of metastatic behavior, treatment and follow-up of melanoma patients.

**Abstract:**

The early detection of cutaneous melanoma, a potentially lethal cancer with rising incidence, is fundamental to increasing survival and therapeutic adjustment. In stages II–IV especially, additional indications for adjuvant therapy purposes after resection and for treatment of metastatic patients are urgently needed. We investigated whether the fatty acid (FA) and protein compositions of small extracellular vesicles (sEV) derived from the plasma of stage 0–I, II and III–IV melanoma patients (*n* = 38) could reflect disease stage. The subpopulation of sEV expressing CD81 EV marker (CD81sEV) was captured by an ad hoc immune affinity technique from plasma depleted of large EV. Biological macromolecules were investigated by gas chromatography and mass spectrometry in CD81sEV. A higher content of FA was detectable in patients with respect to healthy donors (HD). Moreover, a higher C18:0/C18:1 ratio, as a marker of cell membrane fluidity, distinguished early (stage 0–I) from late (III–IV) stages’ CD81sEV. Proteomics detected increases in CD14, PON1, PON3 and APOA5 exclusively in stage II CD81sEV, and RAP1B was decreased in stage III–IV CD81sEV, in comparison to HD. Our results suggest that stage dependent alterations in CD81sEV’ FA and protein composition may occur early after disease onset, strengthening the potential of circulating sEV as a source of discriminatory information for early diagnosis, prediction of metastatic behavior and following up of melanoma patients.

## 1. Introduction

Extracellular vesicles’ (EV) characteristics change depending on the status of the releasing cells. EV comprise different subfamilies, including exosomes, which are lipid bilayer-surrounded endosomal-derived vesicles of 30–150 nm. Cancer impacts the host’s body at different levels, and circulating EV may reflect the actual disease status [1]. Thus, EV monitoring represents a promising non-invasive strategy to obtain useful information on the appropriate diagnosis, progression and response or resistance to therapy [2,3]. Proteomic analysis of serum EV has demonstrated the feasibility of subtyping breast cancer providing biomarkers for early detection and prediction of recurrence risk [4]. Additionally, the integrin landscape of tumor EV allows anticipating the metastatic behavior of their originating cells [5]. EV derived from melanoma have been ascribed a variety of tumor-promoting features with detrimental consequences for the tumor-bearing host. They foster progression, immune suppression and epithelial-to-mesenchymal transitioning; promote the establishment of premetastatic niches; and counter anti-cancer therapies [6]. In contrast to that of healthy donors (HD), the plasma of melanoma patients contains higher amounts of EV with melanoma inhibitory activity (MIA) and calcium binding protein B (S100B), together with CD63 and Caveolin-1 [7,8]. Cutaneous melanoma is an aggressive cancer that tends to early metastasis, and it is increasing in incidence worldwide. In advanced stages, despite the advent of targeted and immunotherapies, the prognosis remains poor, generally, due to tumor immune evasion and intrinsic cell resistance [9,10,11]. Surgery is the treatment of choice in early stages, but advanced disease requires systemic therapy [12]. Tumor thickness, ulceration, invasion of blood vessels or lymphatics and immune response are the predominant markers with which to determine prognosis [13]. Tumor, nodes, metastasis (TNM) classification and stage grouping criteria facilitate risk stratification to guide patient treatment [14]. Early detection of melanoma and its metastases plays a key role in increasing survival and therapeutic adjustment. This is particularly important in stages II–IV for adjuvant therapy purposes after resection, and for treatment of metastatic patients. Several molecules have been proposed as prognostic markers: S100B, MIA, LDH and mutated BRAF [15,16]. Cell-free nucleic acids and circulating tumor cells, detectable in 29–72% metastatic patients, are prognostic for overall survival (OS) [17]. Additionally, PD-L1 immune checkpoint, exposed by tumor and non-tumor EVs, has been tested as biomarker of response to immunotherapy with PD-1 checkpoint inhibitors [18]. In contrast, studies quantifying lipid species in body fluid EV derived from cancer patients are only starting to emerge, as shown for prostate cancer [19]. Lipids are a large group of metabolites that differ in terms of their fatty acid (FA) compositions. FA can be classified into three groups: saturated FA (SFA), without double bonds in the acyl chain; monounsaturated FA (MUFA), with one double bond; and polyunsaturated FA (PUFA), with more than one double bond in the acyl chain. Serum, plasma or erythrocyte membrane MUFA and SFA levels reflect endogenous de-novo FA synthesis. Alterations of lipid metabolism are currently considered a hallmark of many malignancies [20]. Tumor cells possess and acquire numerous strategies to resist the immune system and anticancer therapies. Among these, alterations of the tumor cell lipid metabolism and the consequent abnormal cell membrane composition play prominent roles [21,22]. A growing number of studies have analyzed the relations between lipids and various malignancies, including breast [23], prostate [24,25], ovarian [26], hepatocellular [27], lung [28], pancreatic [29] and bladder [30] cancers. The investigation of metabolic alterations in cancer cells may, on the one hand, provide useful indications about disease stage and progression, and on the other hand, generate knowledge for the development of therapies targeting involved lipid metabolic pathways [31]. Interestingly, cell membrane composition has been studied for decades, but membrane manipulation as a part of cancer therapy remains understudied, especially for melanoma. In this regard, one aspect that can be easily assessed is how membrane fluidity changes during cancer progression. It is known that cancer cells’ lipid compositions may fluctuate over time, depending on their environmental conditions [27,32]. For instance, cells preparing for metastasis reduce their membrane cholesterol content to increase membrane fluidity and plasticity, which is essential for penetrating blood vessels [33]. Unsaturated lipids, such as oleic acid, determine the decrease of membrane lipid packing, consequently improving fluidity. In this context, the saturation index, SI, and the C18:0/C18:1 ratio, also considered a marker of cell membrane fluidity, gain significance [34,35]. In some epidemiological studies, the ratio of MUFA to SFA has been used to reflect stearoyl-CoA desaturase (SCD) activity as a predictive marker of breast cancer risk [36,37].

Small EV (sEV), including exosomes, are enriched in lipids, such as sphingolipids, cholesterol and phosphatidylserine, and display a higher level of saturation in the fatty acyl groups of their phospholipids, with respect to their originating cells. Their composition is similar to lipid rafts, and exosomes have higher lipid order and detergent stability levels than other EV types [38]. Exosomal lipid profiles may also reflect their phenotypes and functions [39,40]. EV can affect lipid metabolism, including the synthesis, transportation and degradation of lipids. EV-mediated lipid metabolism disorders contribute to the generation and progression of diseases, such as cancer, atherosclerosis, non-alcoholic fatty liver disease, obesity and Alzheimer’s disease. Of note, lipid metabolism can also influence the production and secretion of EV, and their interactions with recipient cells. Therefore, EV may represent effective targets for diagnosis and treatment in this context [41].

We combined FA and proteomic studies to investigate potential differences in the plasma sEV profiles of melanoma patients at different stages (0–IV), and compared them to HD. In particular, we focused on immunoaffinity-captured CD81-expressing sEV (CD81sEV) to investigate whether they could harbor potential discriminatory information for early diagnosis and prediction of metastatic potential. We also aimed at understanding whether the proteome and FA compositions of circulating melanoma patients’ CD81sEV might reflect potential tumor-related alterations at the liquid biopsy level.

## 2. Materials and Methods

### 2.1. Human Subjects

Blood was obtained from fasting melanoma patients (*n* = 38), stages 0–IV (Table S1), and from age and gender matched healthy donors (HD = 17) who routine donated blood at Centro Trasfusionale Universitario of Azienda Policlinico Umberto I, Sapienza University of Rome, Italy. Subjects signed informed consent approved by Centro Trasfusionale Universitario and Clinica Dermatologica of Azienda Policlinico Umberto I, Sapienza University of Rome, Italy (board resolution approval number #35/2017). Heparinized blood was diluted 1:2 with PBS1X, centrifuged at 1200× *g* for 20 min, and plasma was collected and stored at −80 °C.

### 2.2. Total sEV Isolation

Total sEV were isolated as described, with modifications [42]. Briefly, the plasma was centrifuged for 30 min at 500× *g* and 45 min at 12,000× *g* (to discard large EV), 0.45-μm filtered (Sartorius, Germany) and ultracentrifuged for 2 h at 110,000× *g* at 10 °C (Sorvall WX Ultra Series centrifuge, F50L-2461.5 rotor, Thermo Scientific, Darmstadt, Germany). The pellet was suspended in PBS, ultracentrifuged at 110,000× *g* for 90 min and preserved for subsequent analyses.

### 2.3. Immunocapture of CD81sEV

To investigate a homogeneous EV population and exclude lipoprotein contamination, we captured sEV expressing CD81 pan EV marker from plasma, which was depleted from large EV, i.e., after the centrifugation at 12,000× *g* for 45 min (see Section 2.2). Briefly, Protein A + G Sepharose resin (Pierce, Thermo Fisher Scientific, Waltham, MA, USA) was washed twice with sodium tetraborate 0.1 M pH 9.0 and incubated with monoclonal anti-CD81 antibody (mAb B-11, Santa Cruz Biotechnology, Heidelberg, Germany) under rotation at RT. After washes, the mAb-CD81-resin was resuspended in 0.1 M sodium tetraborate containing 20 mM dimethyl pimelimidate DMP (Pierce) crosslinker and incubated o.n. To capture CD81sEV, after washing twice with 50 mM Tris pH 7.5, the mAb-CD81-resin was incubated o.n. with plasma.

### 2.4. Electron Microscopy

Transmission (TEM), scanning (SEM) and immune electron microscopy were performed on total sEV purified from 2 mL of plasma [42,43]. CD81 and tsg101 proteins were detected using B11 mAb (Santa Cruz) and polyclonal anti-tsg101 antibody (ab70974, Abcam, Cambridge, UK). Grids were observed by PHILIPS EM208S transmission electron microscope (FEI—Thermo Fisher Scientific, Darmstadt, Germany).

### 2.5. Nanoparticle Tracking Analysis

Total sEV were subjected to nanoparticle tracking analysis (NTA, NanoSight NS300, Malvern Panalytical, Malvern, UK). Detection conditions: capture level 15, threshold 5, slider gain 366, capture duration 60 s. Five videos of 60 s were recorded and analyzed by NTA 3.0 software (Malvern Instruments). 

### 2.6. FA Gas Chromatography Analysis of Total sEV

Starting from total sEV, isolated from 2 mL of plasma/sample as described in Section 2.2, lipids were transmethylated with boron trifluoride-methanol solution (Sigma-Aldrich, Burlington, MA, USA) at 70 °C. Fatty acid methyl esters (FAME) were extracted and analyzed by gas chromatography (Agilent, Palo Alto, CA, USA), equipped with a fused silica capillary column (Omegawax 250, 30 m × 0.25 mm i.d. and 0.25 μm film thickness, Supelco, Sigma-Aldrich) and a flame ionization detector. The injector (split 5:1) temperature was 260 °C, and the detector temperature was set to 280 °C. The heating program began at 190 °C, increased by 2 °C per minute and was held at 240 °C. FAME were identified by comparison with authentic standards (Supelco) and calculated as percentages of total FA. Data were analyzed by Prism (Graphpad, La Jolla, CA, USA).

### 2.7. FA Gas Chromatography-Mass Spectometry Analysis of CD81sEV

Starting from CD81sEV immunocaptured from 2 mL of 12,000× *g* pre-centrifuged plasma, as described in Section 2.3, membrane phospholipids were extracted and transmethylated [44]. FA methyl esters were measured with an Agilent 7890B gas chromatograph coupled to a 5977B quadrupole mass selective detector (Agilent Technologies, Palo Alto, CA, USA). Separations were carried out with an Agilent HP5ms fused-silica capillary column (30 m × 0.25 mm i.d.) coated with 5%-phenyl-95%-dimethylpolysiloxane (film thickness 0.25 μm) as the stationary phase. Injection mode: splitless at a temperature of 280 °C. Column temperature program: 120 °C (1 min), then to 320 °C at a rate of 20 °C/min and held for 10 min. The carrier gas was helium at a constant flow of 1.0 mL/min. The spectra were obtained in the electron impact mode at 70 eV ionization energy; ion source 280 °C; ion source vacuum 10-5 Torr. MS analysis was performed simultaneously in TIC (mass range scan from m/z 50 to 600 at a rate of 0.42 scans s-1) and SIM mode. Data were analyzed by Prism (Graphpad, La Jolla, CA, USA).

### 2.8. LC-MS/MS Analysis of CD81sEV

Electrophoresis was performed on NuPAGE 4–12% (Novex, Invitrogen, Carlsbad, CA, USA), by loading 8 µg of CD81sEV composed of a pool of 2 melanoma patients or 2 HD. For each stage (0–I, II, III–IV), and HD, representing the groups, we analyzed 3 pools, each derived from a pool of 2 subjects, for a total of 6 samples/group. Each gel lane was almost completely cut into 10 slices, avoiding only areas close to 50 and 25 KDa to elude IgG and serum albumin contaminants. Coomassie (Colloidal Blue Staining kit, Invitrogen) stained proteins were enzymatically in gel digested after reduction and alkylation of cysteine residues [45]. The peptide mixture was desalted on a trap column (Acclaim PepMap 100 C18, Thermo Fisher Scientific) and separated on a 20-cm-long silica capillary (Silica Tips FS 360-75-8, New Objective), packed in-house through a reverse-phased capillary high pressure liquid chromatography using an UHPLC nano system (Ultimate 3000 Dionex, Thermo Fisher Scientific), with a 75 min long acetonitrile gradient with a flow rate of 250 nL/min [42]. Eluted peptides were in-line introduced to an Orbitrap Fusion Tribrid Mass Spectrometer (Thermo Fisher Scientific, CA, USA). Orbitrap detection was used for MS1 measurements, at resolution of 60 K (at m/z 200), and MS2 was performed in the ion trap with a rapid scan rate. The most abundant quadrupole isolated precursors were selected in the range of m/z 350–1550, and a data-dependent MS/MS analysis was performed in top speed mode with a 3 s cycle time. Precursors were fragmented with HCD using 32% of normalized collision energy, and dynamic exclusion was enabled for 60 s. A standard Automatic Gain Control (AGC) with 50 ms of maximum injection time was used for MS1, and 20% of normalized custom AGC with a dynamic maximum injection time mode was applied for MS2.

### 2.9. LC-MS/MS Data Analysis

MS raw files were analyzed by Proteome Discoverer 2.4 software (Thermo Fisher Scientific) and peak lists were searched against the human database from UniProtKB/Swiss-Prot database (Release 25 October 2017; 42,253 sequences) by Sequest HT search engine. Peptide identification was obtained using the precursor and fragment tolerances of 10 ppm and 0.6 Da, respectively. Cysteine carbamidomethylation was used as fixed modification, and methionine oxidation and N-acetylation on protein termini were set as variable modifications; specific trypsin cleavage with two missed cleavages were allowed. False discovery rate was set to 1% and determined by searching a reverse database using Percolator node, based on q values. Label-free quantification was based on precursor intensity using all peptides. The proteins were analyzed by VENN diagram (http://bioinformatics.psb.ugent.be/webtools/Venn/, accessed on 1 March 2021). Enrichment analyses including comparison with Vesiclepedia and Exocarta database (http://www.exocarta.org/, accessed on 1 March 2021) [46] were performed using FunRich 3.1.3 [47,48] http://www.funrich.org (accessed on 1 March 2021). Protein–protein interaction was analyzed by String version 11.0 (https://string-db.org, accessed on 1 March 2021) [49]. MS data were deposited into the ProteomeXchange Consortium via the PRIDE [50,51] partner repository, dataset identifier “PXD024434”.

Proteins were analyzed using the “DEP package” [32] within the Bioconductor project [52], based on the statistical programming language R. After quantification, data underwent: (1) filtering: proteins (*n* = 526) were selected when showing at least the 80% valid values; (2) variance stabilizing transformation for normalization; (3) imputation according to the “minDet” method: replacing the missing entries with the estimated minimal value for in each sample. To identify differentially expressed proteins, the three stages grouped as 0–I, II and III–IV were compared to HD, via combined approach, relying on protein-wise linear models and empirical Bayes statistics [53]. Proteins were marked as significant based on two thresholds: a p-adjusted lower than 0.05 and a fold change higher than 1.5. Correction was applied for multiple hypotheses testing through the algorithm of false discovery rate estimation, as implemented in the “fdrtool” package [54].

### 2.10. Statistics

Statistical analyses were performed using Prism software v.5 (GraphPad, La Jolla, CA, USA) and R version 4.1.0 (Vienna, Austria). Comparisons between continuous variables in two groups were performed using an unpaired two-tailed Student’s *t*-test. Comparisons between continuous variables in three or more groups were performed using a Kruskal–Wallis test and Dunn’s post hoc test. The melanoma survival data in The Cancer Genome Atlas (TCGA) were obtained from cBioPortal database (http://www.cbioportal.org, accessed on 1 March 2021). Gene associations with overall survival (OS) and/or progression-free survival (PFS) were first examined with univariable and multivariable Cox’s proportional hazard models. Their joint association with OS and/or PFS was then investigated through building a prognostic score based on the following steps: (a) definition of an optimal cutoff for each selected gene by maxstat algorithm (Maximally Selected Rank Statistics) based on RNA-seq value; and (b) construction of the score, as an enumeration of genes above the cutoff value. The score built on data included 5 genes; hence, we could split the patient cohort into different groups based on possible scores obtained for each case series. Survival curves according to the levels of the selected score were finally estimated with the Kaplan–Meier method. The delta value was calculated as the difference between pre and post for each gene, and boxplots were used to show the differences in the two types of patients responding to therapy [55].

The conventional two-sided 5% level was chosen as the threshold of statistical significance.

## 3. Results

### 3.1. Morphologies and Concentrations of Total sEV of Melanoma Patients and HD 

**Figure 1:**
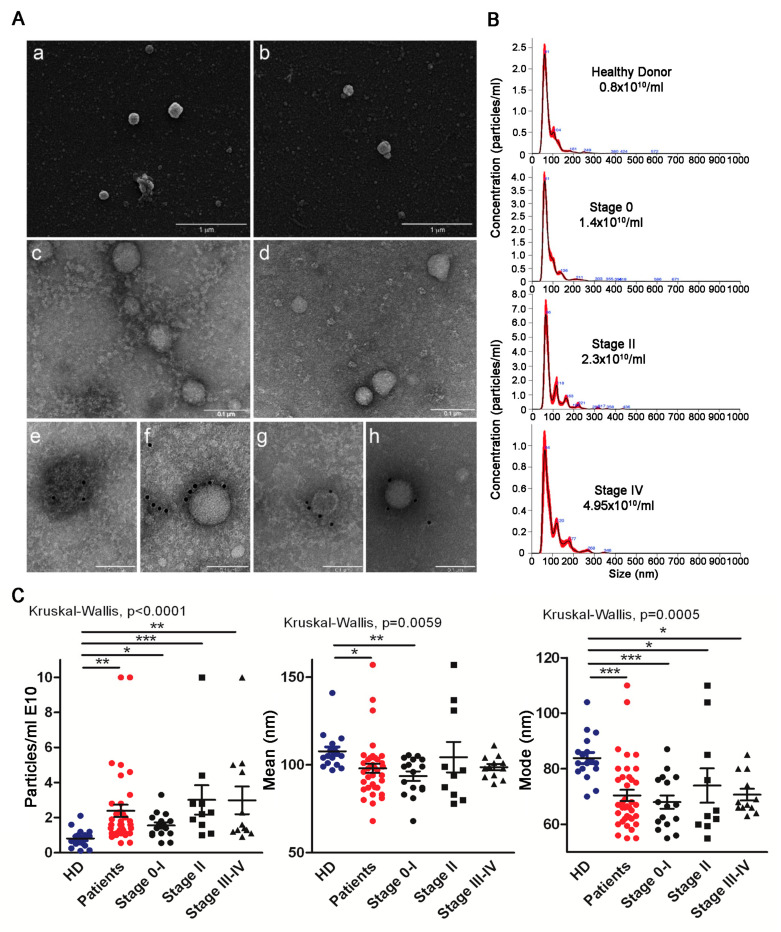
Morphology and nanoparticle tracking analysis (NTA) of total sEV. (**A**) Scanning electron microscopy analysis of total sEV from a HD (**a**), and from a stage II melanoma patient (**b**); bars, 1 µm. Transmission electron microscopy analysis of HD sEV (**c**) and stage II patients’ sEV (**d**); bars, 0.1 μm. (**e**–**h**) Immunoelectron microscopy combined with positive/negative contrast method: D sEV (**e**,**f**) and stage II patients’ sEV (**g**,**h**) showing the presence of CD81 (**e**,**g**) and tsg101 (**f**,**h**); bars, 0.1 µm. (**B**) Representative NTA profiles of HD and melanoma patients’ (stages 0, II, IV) sEVs. (**C**) NTA characterization of total sEV from HD and patients, with different stages of disease (particles/mL, mean and mode); HD *n* = 17; patients *n* = 38. Statistical significance was achieved with unpaired *t*-tests for HD vs. patients and with Kruskal–Wallis and Dunn post-tests for HD vs. stages. * *p* < 0.05, ** *p* < 0.001, *** *p* < 0.0001.

Our principal aim was to verify whether circulating sEV derived from the plasma of early stage melanoma patients, including stages 0–I (in situ melanoma) and II, harbored discriminating characteristics with respect to advanced patients (stage III–IV) and in comparison to HD. We thus isolated total sEV by differential centrifugation according to our protocol, which included a 45 min 12,000× *g* centrifugation followed by 0.45 μm filtration to eliminate large sEV prior to a 110,000× *g* ultracentrifugation step. SEM (Figure 1A, panels a and b) and TEM (Figure 1A, panels c and d) showed the presence of vesicles displaying the typical structure and dimensions of exosomes, as depicted by representative HD (Figure 1A, panels a and c) and melanoma stage II sEV (Figure 1A, panels b and d). The expression of CD81 and tsg101 proteins confirmed their exosomal-like nature (Figure 1A, panels e and f HD; g and h patient CD81 and tsg101, respectively). NTA profiling highlighted differences in particle concentration and size (mode and mean), as shown by the representative sEV spectra of a HD and patients with different stages of disease (Figure 1B,C). Of note, the number of circulating sEV augmented with progression, as shown by the significant differences between HD and patients, and between HD and the different stages. Stage II sEV displayed the greatest (statistically significant) difference compared to HD sEV (Figure 1C, left panel). NTA also revealed that total sEV of patients were significantly smaller (patients’ sEV’ mean 93.8 +/− 9.4; mode 67.2 +/− 8.2) than those of HD (HD sEV’ mean 107.7 +/− 10; mode 83.8 +/− 8.0). The difference was particularly evident for the sEV mode, which was significantly lower in all stages with respect to HD (Figure 1C, right panel). The largest significant difference was between HD and stage 0–I CD81sEV.

### 3.2. FA Profiles of Total and CD81sEV 

**Figure 2:**
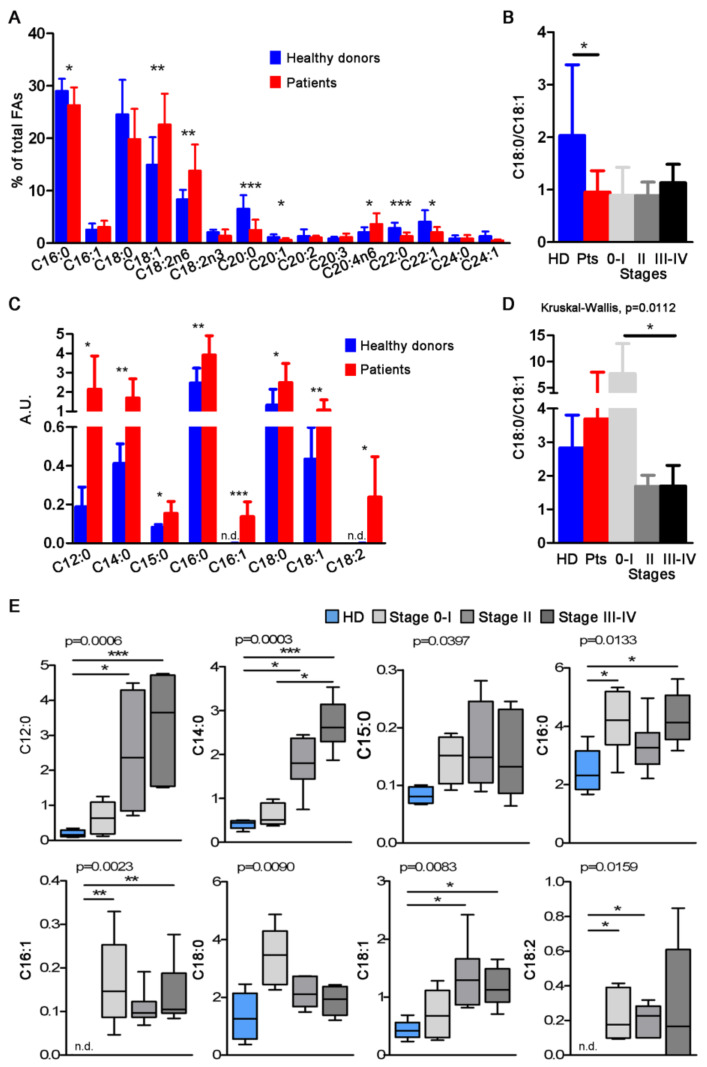
Fatty acids in total sEV and CD81sEV. (**A**) Analysis by gas chromatography of FA in total sEV from HD and melanoma patients. (**B**) C18:0/C18:1 ratio of the samples in A. (**C**) Gas chromatography and mass spectrometry analysis of FA in CD81sEV from HD and patients. (**D**) The respective C18:0/C18:1 ratios of the samples in C. (**E**) Single FA of stage 0–I, II, III–IV and HD in CD81sEV. n.d. = not detected. Statistical significance was achieved with unpaired *t*-tests for HD vs. patients and with Kruskal–Wallis and Dunn post-tests for HD vs. stages. * *p* < 0.05, ** *p* < 0.001, *** *p* < 0.0001.

Gas chromatography analysis of total sEV showed a prevalence of saturated FA, essentially palmitic (C16:0) and stearic acids (C18:0). Compared to HD, patient total sEV displayed a general decrease of saturated FA (C16:0, C20:0, C22:0) and an increase of unsaturated FA (C18:1, C18:2n6, C20:4n6) (Figure 2A). Despite the difference in C18:0 not being significant between HD and patients, the increase in C18:1 in patients’ sEV sufficed to reach statistical significance in the C18:0/C18:1 ratio (Figure 2B). At the single FA level, total sEV derived from HD and the different melanoma stages displayed differences for C18:1, C18:2n6, C18:2n3, C20:0, C22:0 and C22:1 FA (Figure S1). Capturing CD81sEV led to the loss of long/very long chain FA, revealing a different profile with respect to total sEV, while evidencing the presence of C12:0 and C14:0 saturated FA that were not detectable in total sEV (Figure 2C). For all detected FA, patients displayed higher contents compared to HD, which was particularly evident for C12:0 lauric, C14:0 myristic, C16:0 palmitic, C18:0 stearic, C18:1 oleic and C18:2 linoleic acids (Figure 2C). Interestingly, the trends of C16:0 and C18:0, the most abundant FA in nature, were inverted in total sEV compared to CD81sEV, and C18:1 was the only coherent FA, displaying increased levels in patients compared to HD in both total and CD81sEV (Figure 2A,C). The C18:0/C18:1 ratios of CD81sEV were similar among HD and all patients, but significantly different among the individual stages and HD (*p* = 0.0112 Kruskal–Wallis). In particular, the ratio was reduced in stage III–IV CD81sEV with respect to those derived from stage 0–I patients (Figure 2D). At the single FA level, the statistical analysis of CD81sEV from HD and the stages 0–I, II and III–IV revealed differences via Kruskal–Wallis test for all FA (Figure 2E). With respect to donors’ CD81sEV, we could also measure increased expression in stages II and III–IV of C12:0 and C14:0, although these FA are usually expressed at very low levels in human cells, and of C18:1 (Figure 2E). Additionally, the expression of C14:0 was significantly increased in stage III–IV CD81sEV compared to stage 0–I ones (Figure 2E), representing the only difference we could detect among the different stages. In contrast, C16:1 and C18:2 were undetectable in CD81sEV from HD (Figure 2C,E). Of note, stage 0–I CD81sEV displayed increased C16:0, C16:1 and C18:2 with respect to HD (Figure 2E).

### 3.3. Proteomic Analysis of CD81sEV

**Figure 3:**
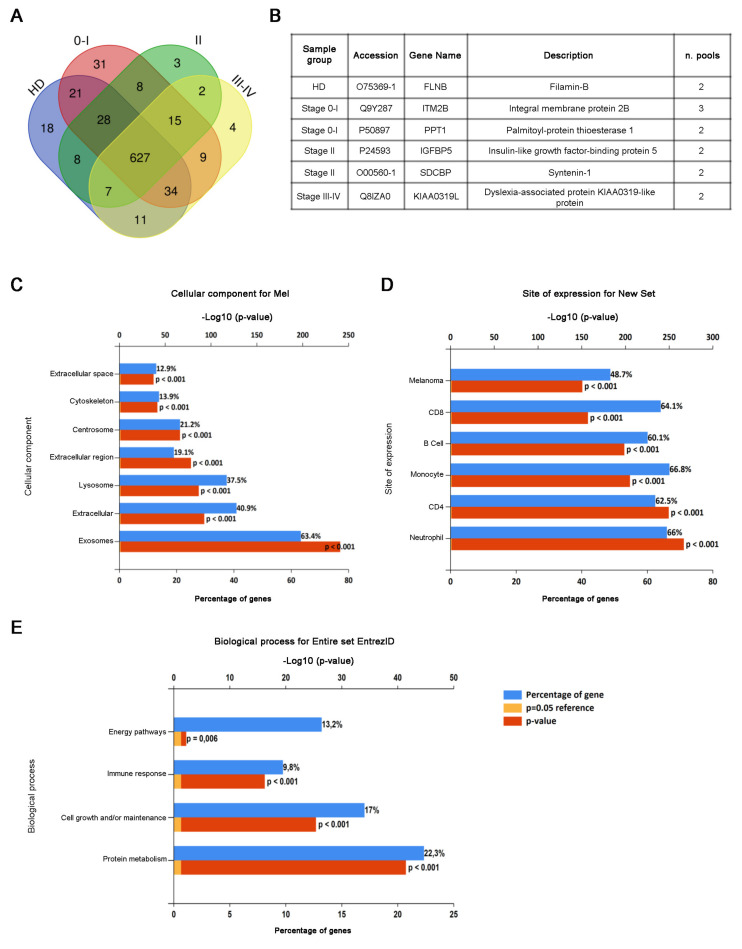
Proteomic analysis of CD81sEV. (**A**) A Venn diagram representing the number of proteins uniquely detected in each HD, stage 0–I, stage II or stage III–IV group or shared among two, three or four-sample groups. (**B**) A table of proteins identified at least in two pools uniquely in one sample group. (**C**–**E**). Enrichment analysis of identified proteins by FunRich analysis for cellular component (**C**) site of expression (**D**) and biological process (**E**). Blue bars represent the percentages of protein genes assigned to the indicated terms. Yellow bars show the reference *p* value (0.05), and red bars show the calculated *p* value of enrichment for the indicated term.

CD81sEV of patients of stages 0–I, II and III–IV and HD were analyzed by LC-MS/MS. We identified a total of 899 proteins (Table S2) and quantified 826 of them (data are available in Proteome Xchange with identifier PXD024434) using a label free approach. All the following described analyses concern these 826 proteins, from now on reported as “detected proteins”. We found small differences among the numbers of the proteins detected in the biological replicates (Figure S2A). An average of 631 proteins was detected in each sample group (Figure S2B). Among the proteins we quantified, we found LDH, a marker of a melanoma [56], which displayed a gradual but not significant increase, which peaked in stage III–IV CD81sEV. Similarly, members of the S100 protein family involved in inflammation and cancer displayed higher-intensity mass spectrometry signals in stage 0–I CD81sEV compared to HD (Table S2), although not statistically significantly higher [57]. The biggest portion (average 87%) of the proteins was detected at least in two independent pools (Figure S2C,D). The Venn diagram (Figure 3A) shows that few proteins were uniquely detected in just one sample group (healthy or melanoma stage), and almost all of these were identified exclusively in one pool. Only six proteins were identified in at least two pools uniquely in one specific sample group (Figure 3B): filamin B for HD; integral membrane protein 2B and palmitoyl-protein thioesterase 1 for stage 0–I; insulin-like growth factor-binding protein 5 and syntenin-1 for stage II; and dyslexia-associated protein KIAA0319-like protein for stage III–IV. In total, 97% and 95% of the detected proteins were present in the entire and specific exosome Vesiclepedia databases, respectively (Figure S2E,F), and 81% were among the top 100 Vesiclepedia proteins (Figure S2G). Similarly, 83% of the proteins present in our dataset were already in the Exocarta database (Figure S2H). Cellular component analysis also showed the highest enrichment in exosomes (Figure 3C), confirming the good quality of our preparations. The comparison of our protein set with the FunRich database of protein expression sites evidenced significant enrichments in melanoma and immune cells, including neutrophils, monocytes, B cells and CD4 and CD8 T cells (Figure 3D). The analysis of the biological processes involving the detected proteins showed significant enrichments, particularly in relation to protein metabolism, cell growth and/or maintenance and the immune response (Figure 3E).

**Figure 4:**
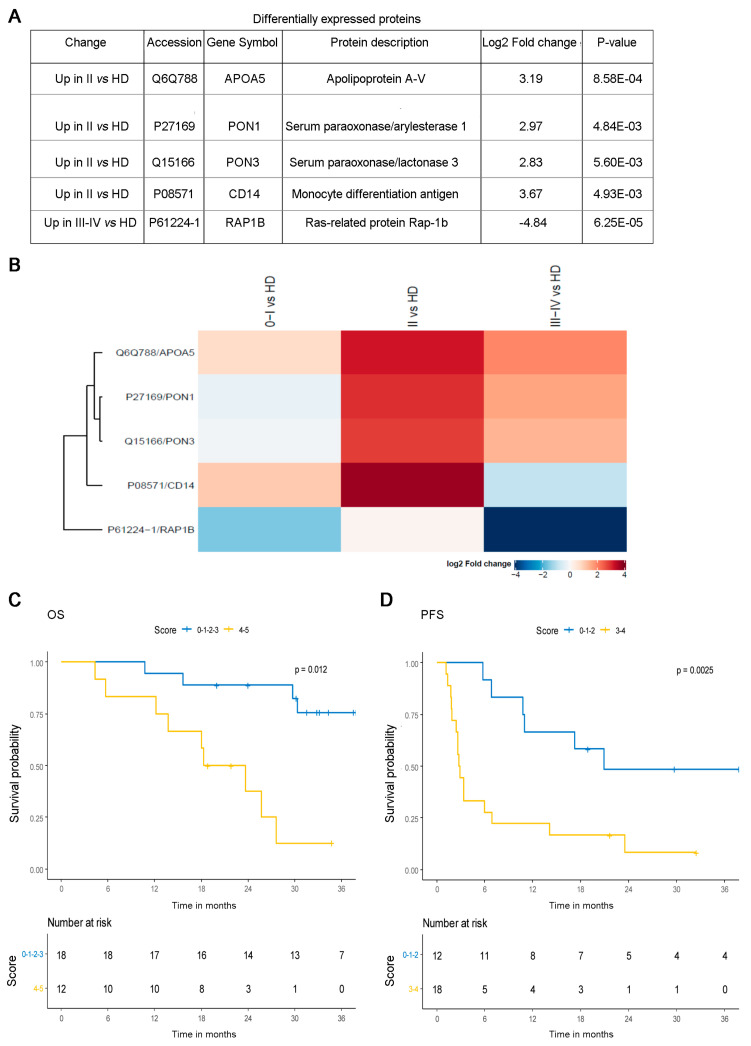
Analysis of differentially expressed proteins in CD81sEV. (**A**) Table of proteins found as statistically differentially expressed in CD81sEV from HD versus patients. (**B**) Protein fold changes in the different stages. The proteins APOA5 (Q6Q788), PON1 (P27169), PON3 (Q15166) and CD14 (P08571) were increased in stage II in comparison with HD; RAP1B (P61224-1) was decreased in stage III–IV versus HD. C,D. Overall survival (OS) (**C**) and progression-free survival (PFS) (**D**) of metastatic melanoma patients (baseline levels of transcripts detected by RNA-seq in plasma EV from Shi et al. [55], validation dataset, *n* = 30), based on the expression levels of the 5 genes assessed by multivariable score approach. Patients with low scores (≤3 or >3 altered genes for OS and ≤2 or >2 altered genes for PFS) had significantly better prognoses. Kaplan–Meier survival curves with log-rank P values are shown.

The quantified proteins were analyzed by the DEP package in order to recognize significantly different protein abundances. Five proteins were found to be significantly differentially expressed (Figure 4A). APOA5, PON1, PON3 and CD14 were overexpressed in stage II CD81sEV in comparison with HD (Figure 4B). The protein RAP1B was underexpressed in stage III–IV patients with respect to HD. We detected the largest difference between HD and stage II. Protein–protein interactions were analyzed by STRING (Figure S3), and three proteins, PON3, PON1 and APOA5, displayed direct interactions. Two biological processes were enriched (FDR < 0.05): PON1 and PON3 are involved in the lipoxygenase pathway; and APOA5, RAP1B and CD14 are involved in the regulation of vesicle-mediated transport. 

To explore whether the identified proteins, APOA5, PON1, PON3, CD14 and RAP1B, could be of any clinical relevance, we evaluated their expression in silico in relation to the available clinical information in the TCGA dataset of primary melanoma samples (PM, *n* = 80). When we determined the expression of APOA5, PON1, PON3, CD14 and RAP1B in PM, we noticed that APOA5 and PON1 were undetectable in the majority of tissue samples, as these proteins are principally found in the blood. We thus decided to exclude APOA5 and PON1 from the analysis. To evaluate the relationship among PON3, CD14 and RAP1B and prognoses of melanoma patients, we conducted Kaplan–Meier survival analysis according to gene expression data of PM (Figure S4A). By estimating the joint contribution of the three genes, we were able to divide patients into two groups. Regarding OS, by pooling low scores (0–1; consisting of 0 or only 1 altered gene) versus high scores (>1; having more than 1 altered genes), we observed that the low scores were associated with significantly (*p* = 0.0027) better OS than the high scores (Figure S4B). To explore the clinical relevance of APOA5 and PON1 as well, we selected a dataset containing RNA-seq data from circulating plasma-derived EV of metastatic melanoma patients (55). By considering all five selected genes, we could cluster patients (*n* = 30) into two groups on the basis of the score ≤3 or >3 altered genes (Figure 4C). It is interesting to note that the low scores were associated with a significantly better prognosis (*p* = 0.012), in accordance with the TCGA data. Considering PFS, we could obtain similar results by dividing the two groups according to the scores ≤2, or >2 altered genes (Figure 4D). In particular, at the single gene level, low levels of expression of APOA5, PON1, PON3 and CD14 associated with better prognosis, and in contrast, low expression of RAP1B was associated with worse prognosis, supporting our proteomics data showing a significant decrease in CD81sEV in stage III–IV melanoma patients (Figure S5A,B and Figure 4A). Of note, survival analysis by dividing patients into responders and non-responders to treatment with ICI (validation cohort of [55]; *n* = 30) revealed a statistically significant increase (*p* = 0.0288) of APOA5 in responding patients, as shown by the delta between pre and on-treatment gene expression values. A similar trend was observed for the remaining genes (Figure S5C).

## 4. Discussion

Despite representing less than 5% of all cutaneous malignancies, melanoma accounts for the majority of skin cancer deaths [58]. Although the advent of targeted and immune therapies has improved the median survival of metastatic patients, factors informing the surveillance and early detection of initiation and progression are urgently needed to guide clinical interventions. Notably, in this context, EV have generated enthusiasm due to their potential utility in liquid biopsies [59,60]. Plasma-derived EV from cancer patients could reflect in part the molecular and genetic contents of the releasing parent tumor cells, and thus their protein contents may be informative [61]. Similarly, lipid metabolic alterations of tumor cells have attracted major interest [62]. In fact, lipids represent a source of energy and form the membrane’s structural foundation, but can also contribute to disease progression. An increased lipid synthesis rate is one of the most prominent metabolic reprogramming features in melanoma, and various alterations in FA metabolism can sustain melanoma cell aggressiveness. Elevated expression of the key lipogenic FA synthase is associated with tumor cell invasion and poor prognosis. Increased FA biosynthesis, due to elevated expression of FA synthase and FA uptake, may increase the levels of multiple lipids with a signaling function that can affect numerous cellular processes, including cell differentiation and motility of melanoma [63]. We and others have previously found that compared to HD, advanced melanoma patients have increased amounts of circulating EV [8], and that the protein amount is higher in late versus early stages. It decreases in response to therapy in breast cancer patients [64]. Here we show that increased sEV concentrations are detectable already in stages 0–II (Figure 1C), and sEV showed a 20% size reduction between HD and patients. In particular, the most significant reduction in mode was evidenced in early stage (0–I) total sEV. Such characteristics may facilitate EV distribution to lungs and crossing the blood–brain barrier, promoting pre-metastatic niche formation [65]. On the other hand, we cannot rule out that the increase in concentration of total sEV, isolated as bulk sEV by differential centrifugations, might have resulted from lipoproteins precipitating during the ultracentrifugation step, despite our isolation protocol including a 12,000× *g* centrifugation to reduce such contaminations [66]. Similarly, total sEV contained long and very long-chain FA (Figure 2A) that were not detectable in CD81sEV (Figure 2C), potentially derived from lipoprotein contaminants precipitating during total sEV isolation by ultracentrifugation [66,67]. With respect to total sEV, FA analysis of CD81sEV revealed a lower number of FA species, which were all more abundant in patients’ CD81sEV with respect to HD and included C12:0 lauric, C14:0 myristic, C15:0 pentadecanoic, C16:0 palmitic, C16:1 palmitoleic, C18:0 stearic, C18:1 oleic and C18:2 linoleic acids (Figure 2C). C16:1 and C18:2 were detectable only in CD81sEV derived from patients but not in those from HD. This could be explained by our selection of the CD81-expressing EV population, which was increased and thus detectable in cancer patients’ CD81sEV, or by the higher levels of these two FAs in the CD81sEV, as shown in the sera of colorectal cancer patients [68]. An association of cancer with serum FA alterations in the early stages with respect to HD has been shown for breast cancer patients, where the FA synthase expressed at the tumor site could influence serum FA composition [69]. Upon comparing patients’ FAs in total sEV with those detected in CD81sEV, C18:1 was the only coherent FA, in that it was increased in both total sEV and in CD81sEV. This may be related to the fact that C18:1 is the most abundant unsaturated FA in lipid membranes. C18:1 was also significantly increased in stage II and III–IV CD81sEV. This was reflected by the C18:0/C18:1 ratio, or SI, which decreased significantly in stage III–IV patients, with respect to stage 0–I, suggesting a link with disease progression (Figure 2D). The SI has been proposed as a malignancy marker, consistently with its reduction in neoplastic cells and in red blood cell membranes of cancer patients [34,35,70]. Decreases in the C18:0/C18:1 ratio have been observed in colorectal cancer [71], bronchogenic carcinoma [72], lymphoma [73], leukemia [74], malignant liver neoplasms [75] and in gallbladder cancer [35]. In this context, our results support that the SI might be considered a diagnostic marker of malignancy, and the FA profile changes could provide the first signs of disease. In contrast to total sEV, the sFA levels in CD81sEV showed that with respect to HD, all detected FAs were substantially increased, and this was reflected also by the single stages (Figure 2C,E) with respect to HD, highlighting the important differences in SI 0–I early and late melanoma stages. The higher levels of lauric (C12:0) and myristic (C14:0) acids in stage II and III–IV CD81sEV may be related to the accelerated metabolism of progressing and advanced cancer [76]. Of note, as the only FA significantly different between the stages, C14:0 was overexpressed in stage III–IV compared to stage 0–I, suggesting an association with melanoma progression. High serum levels of free C14:0 were also identified as an indicator for systematic progression of pre-leukemic conditions towards acute myeloid leukemia [77]. Moreover, C14:0 increase was related to risk of breast cancer occurrence in a nested case-control study [78]. We showed that stage II and III–IV CD81sEV contained more C18:1 than HD-derived CD81sEV (Figure 2E), and this result is in line with the correlation of C18:1 and the metastatic potential [79]. In fact, the adhesion and migration of tumor cells are linked to higher membrane fluidity, due in turn to an increase of unsaturated FA. Finally, we deemed it advantageous to evaluate FA in a specific EV subpopulation, such as the CD81sEV, with respect to total sEV. In fact, we could detect only 3/15 significant differences in the single FA expression levels of total sEV between groups, in contrast to the 6/8 in CD81sEV. Proteomics of CD81sEV identified 899 proteins, 97% of which were comprehensive of all vesicles and 95% specific for exosomes; and 81% of the top 100 Vesiclepedia proteins were detected in our dataset, and 83% were common to Exocarta, supporting the efficiency of our CD81-capture. Of the proteins identified at least in two pools uniquely in one sample group (Figure 3B), the integral membrane protein 2B (ITMB2), detected in stage 0–I, is involved in p53-independent apoptosis [80]. Palmitoyl-protein thioesterase 1 (PPT1), also identified in stage 0–I, is increased in cancer and associated with poor survival [81]. This suggests that even in very early melanoma, relevant changes in protein composition of circulating CD81sEV may occur, justifying the rare cases of metastatization [82]. Insulin-like growth factor-binding protein 5 (IGFBP5) and syntenin-1 were identified only in stage II CD81sEV. The presence of IGFBP5, a tumor suppressor [83], in circulating sEV, suggests its discharge from cells, likely favoring tumor progression. Similarly, syntenin can support cell proliferation and migration [84], and its presence in sEV could thus mediate tumor growth. When we quantified the proteins detected by mass spectrometry by label free approach, five proteins were significantly and differentially expressed in CD81sEV. APOA5, PON1, PON3 and CD14 were increased in stage II, an interesting finding, since stage II is at the borderline between a tumor in situ (stage 0–I) and distant metastasis (stage III–IV). APOA5, expressed by platelet-derived EV (ExoCarta) and podocyte-specific CR1-immunocaptured urine exosomes, modulates intra/extracellular triacylglycerol metabolism [85,86], and this may relate to the differences measured in FA composition. PON1 is implicated in eliminating carcinogenic lipid-soluble radicals. In esophageal squamous cell carcinoma, increased oxidative stress is associated with decreased antioxidant PON1 activities [87]. PON1 levels may act as an indicator of oxidative stress in cancer [88]. PON3, overexpressed in tumors, promotes cell death resistance. Moreover, PON3 can impair endoplasmic reticulum (ER) stress-induced apoptotic MAPK signaling [89]. Of note, APOA5, PON1 and PON3 displayed a direct interaction in STRING analysis. PON1 and PON3 are involved in the lipoxygenase pathway (Figure S3), leading to the generation of eicosanoids impacting cancer development, progression and immune responses [90]. Finally, of the four proteins increased in stage II CD81sEV, CD14 monocyte marker displayed the highest expression. Myeloid cell alterations, including the accumulation of myeloid-derived suppressor cells, are typical in advanced stages [91]. However, routinely performed blood counts have evidenced survival-associated alterations of peripheral blood leukocytes, including an increase in monocytes, in early-stage melanoma patients [92]. In stage III–IV CD81sEV, we found a decrease in RAP1B expression compared to HD. This was surprising, since RAP1B is involved in MAPK and integrin activation in melanoma, [93], but was also identified as a pan-EV marker [94]. Furthermore, RAP1B is expressed by platelet-derived sEV, and thus is physiologically present in circulating EV [94]. The decrease we measured in CD81sEV may thus potentially depend on the expansion of other EV populations—for instance, those derived from immunosuppressive immune cells accumulating during progression. In fact, we investigated plasma sEV: those proteins increased in stage II CD81sEV may relate to the changes occurring during early stages of cancer, which could be reflected systemically also by plasma EV. The presence of cancer may indeed influence the plasma EV population, as also recently found by Pietrowska et al., who captured melanoma and non-melanoma-derived EV in the plasma of 15 patients, comprising also stage I–II patients at diagnosis. According to their data, in plasma of these patients they could also capture melanoma antigen CSPG4, indicating the presence of melanoma-derived EV at the systemic level, despite the early stage of disease [61]. Thus, we are confident that our results—although gathered from a small cohort and not validated, a limitation of our study—may nonetheless advance the field, even if still far from translation into the clinical practice. Nonetheless, EV-based biomarkers can be used for routine clinical diagnosis, as shown for the exosome-based ExoDx Prostate (IntelliScore) EPI test [95]. The importance of EV for disease markers and as intercellular communicators also depends on their nucleic acid contents, including single and double-stranded DNA, mitochondrial DNA, mRNA and microRNA [96,97]. In breast cancer brain metastasis, patients’ microRNAs carried by circulating EV function as distinctive biomarkers of precocious and advanced stages [98]. In melanoma cells it has been demonstrated that in hypoxic conditions, EV exhibit a miRNome and proteome signature (AKR7A2, DDX39B, EIF3C, FARSA, PRMT5, VARS), which is associated with poor prognosis for the patients [99]. By proteomics we identified five proteins in plasma CD81sEV that were differentially expressed between HD and stage II (APOA5, PON1, PON3 and CD14) and between HD and stage III–IV, advanced stage melanoma, (RAP1B). Of these, PON3, CD14 and RAP1B were evaluated for their association with survival in the primary melanoma sample data set of the TCGA. Kaplan–Meier curves showed their prognostic relevance upon joint modeling as prognostic score in OS. These results may suggest an association between the tumor and systemic level in relation to CD81sEV. The tumor samples of the TCGA displayed undetectable levels of APOA5 and PON1, as these proteins are found at the systemic level. We thus searched for a dataset derived from circulating EV containing clinical information to evaluate our identified molecules, including APOA5 and PON1. In their transcriptomic analysis of plasma-derived EV of melanoma patients, Shi et al. revealed a correlation with resistance to immune checkpoint inhibitors (ICI) and melanoma progression, and thus a clinical response to ICI [55]. The evaluation of the clinical relevance of our proteins in the dataset of Shi et al. evidenced that those proteins with low scores were associated with a significantly better prognosis, both for OS and PFS. This analysis also evidenced that lower expression levels of APOA5, PON1, PON3 and CD14 were associated with better prognosis. In contrast, reduced levels of RAP1B were associated with worse prognosis of patients, supporting the reduction of RAP1B we detected in stage III–IV melanoma patients’ CD81sEV. Finally, by dividing patients into responders and non-responders, we observed an increase of APOA5 in the plasma EVs of responding patients, supporting the strong interaction between immunotherapy and lipid metabolism [100].

## 5. Conclusions

We propose that the concomitant investigation of FA and protein alterations in plasma CD81sEV could represent a new and useful approach to improve the diagnosis and treatment of cutaneous melanoma, especially in the early stages. Circulating CD81sEV, composed of EV derived from different cell types, including immune and tumor cells, may change depending on the homeostasis of the entire organism, reflecting and corroborating with the concept that “cancer is a systemic disease” [101,102].

## Data Availability

The MS data presented in this study are openly available in the ProteomeXchange Consortium via the PRIDE partner repository, dataset identifier “PXD024434”.

## Supplementary Materials

The following are available online at https://www.mdpi.com/article/10.3390/cancers13164157/s1, Table S1: Clinicopathological features of melanoma patients; Table S2: List of all the proteins identified in each melanoma stage or healthy donor; Figure S1: Fatty acids in total sEV; Figure S2: Proteomic analysis of CD81sEV; Figure S3: Protein-protein interaction analysis by STRING; Figure S4: Association between the 3-gene signature and overall survival of PM patients (TCGA); Figure S5: Association between the expression of the five genes by circulating EV, survival and clinical response [55].
