# Supplementary material for: The Fatty Acid and Protein Profiles of Circulating CD81-Positive Small Extracellular Vesicles Are Associated with Disease Stage in Melanoma Patients
Source: Cancers (Basel). 2021 Aug 18;13(16):4157. doi: 10.3390/cancers13164157 (PMC8392159; doi:10.3390/cancers13164157)
Supplement: Supplementary file 1 [file cancers-13-04157-s001.zip › Suppl Files Paolino et al.pdf]

Table S1. Clinicopathological features of melanoma patients

Gender	22 Male	16 Female
Median Age	55,5 (23-89)	
Cutaneous Sites	12 Peripheral	26 Axial
Breslow	1.1 (in situ-3.5)	
Ulceration	12 Presence	26 Absence
SLN*	8 Positive	30 Negative
Stage 0	6	
Stage I ∞	10	
Stage II ∞∞	10	
Stage III ∞∞∞	4	
Stage IV	8	

Anatomic sites of primary tumor: axial site included head, neck, and trunk, while peripheral site included upper and lower limbs; SLN indicates Sentinel Lymph Node; ∞ 7 stage IA and 3 stage IB; ∞∞ 5 stage IIA, 4 stage IIB and 1 stage IIC; ∞∞∞ 3 stage IIIA and 1 stage IIIB.

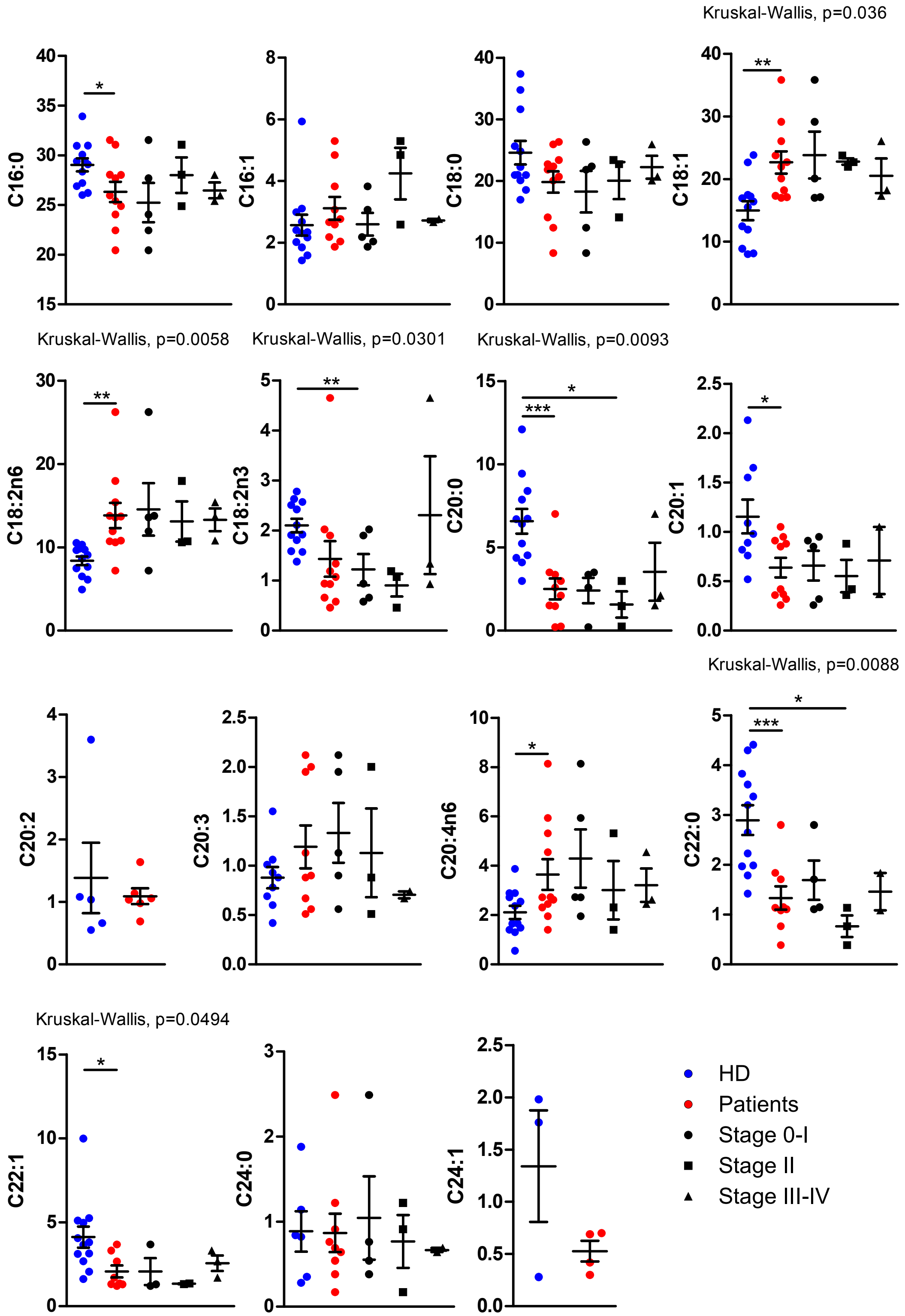

Figure S1. Fatty acids in total sEV. Analysis by Gas Chromatography of FA in total sEV from HD and melanoma patients. Single FA of stage 0-I, II and III-IV vs HD in total sEV. Statistical significance was achieved with unpaired t-test for HD vs patients and with Kruskal-Wallis and Dunn post test for HD vs stages. * p<0.05, ** p<0.001, *** p<0.0001.

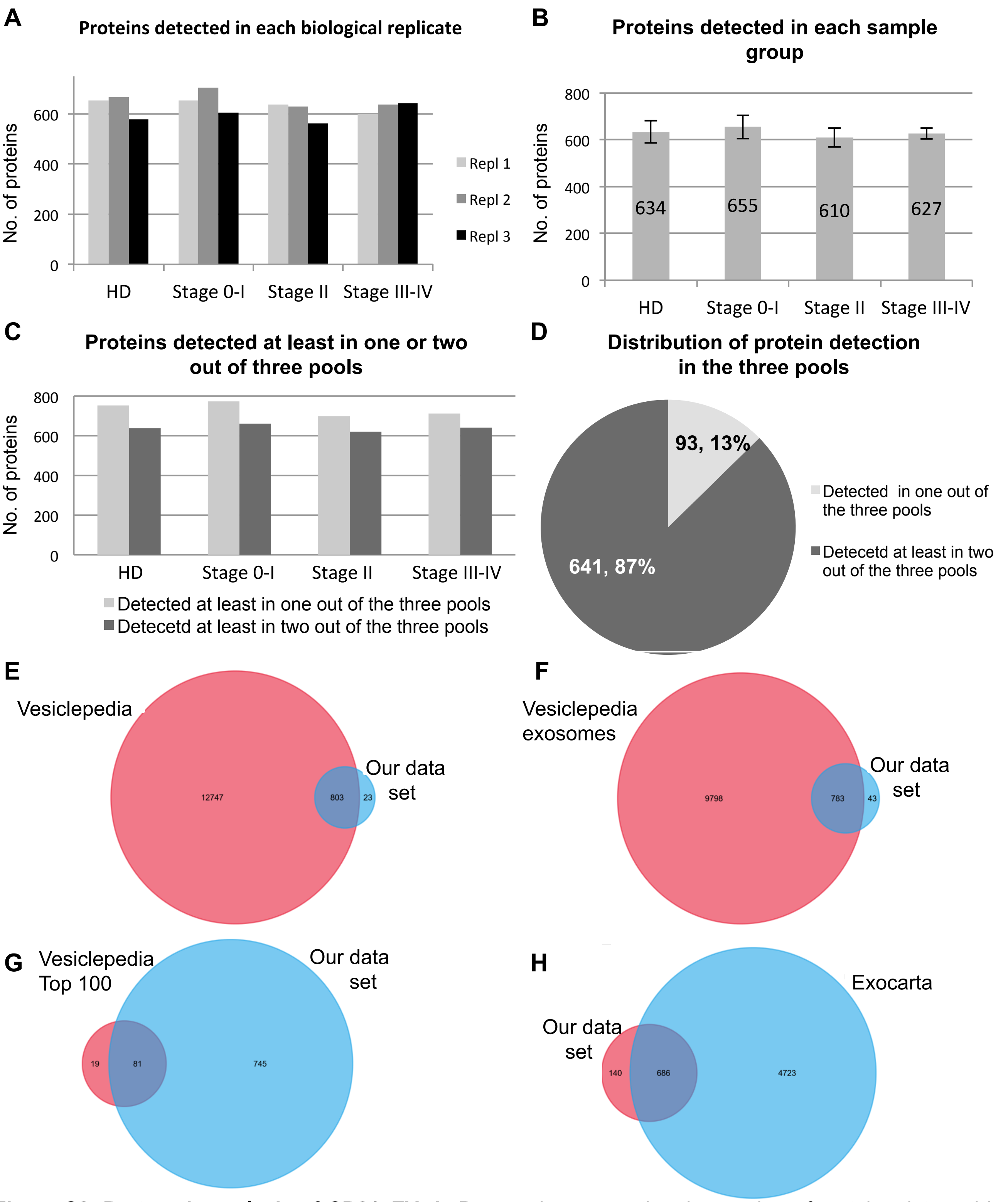

Figure S2. **Proteomic analysis of CD81sEV. A.** Bar graph representing the number of proteins detected in each biological replicate (HD, stage 0-I, stage II, stage III-IV). **B.** Bar graph showing the average number of proteins detected in each sample group, with vertical bars representing standard deviation. **C.** Bar graph displaying the number of proteins detected in at least one or two out of three pools for each sample group. **D.** Percentage of proteins detected in at least two samples (87%) or in one sample (13%). **E.** Comparison of experimental data with Vesiclepedia database comprehensive of all vesicle types using FunRich tool. **F.** Comparison of experimental data with Vesiclepedia-exosome specific database, using FunRich tool. **G.** Comparison of experimental data with the top 100 Vesiclepedia proteins. **H.** Comparison with Exocarta database.

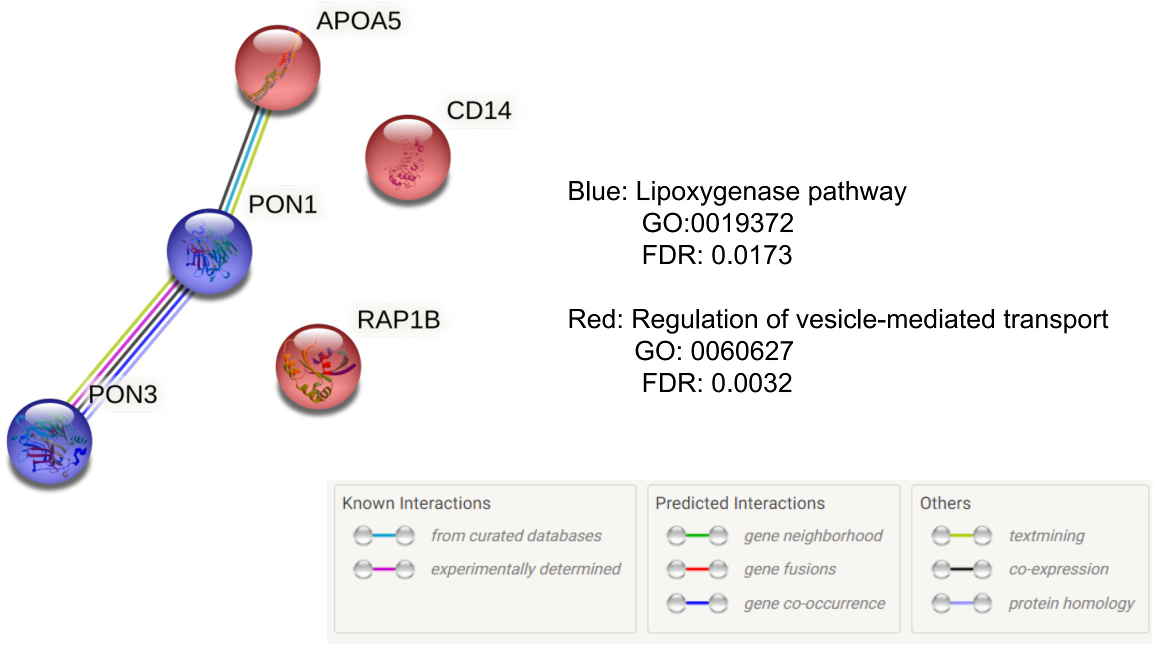

Figure S3. Protein-protein interaction analysis by STRING. STRING analysis of the five differently expressed proteins in CD81sEV. Blue circles belong to Lipoxygenase pathway (GO:0019372), red circles to the regulation of vesicle-mediated transport (GO: 0060627). The relative biological process enrichment FDR and the colored line legend for the nature of protein-protein interactions are reported.

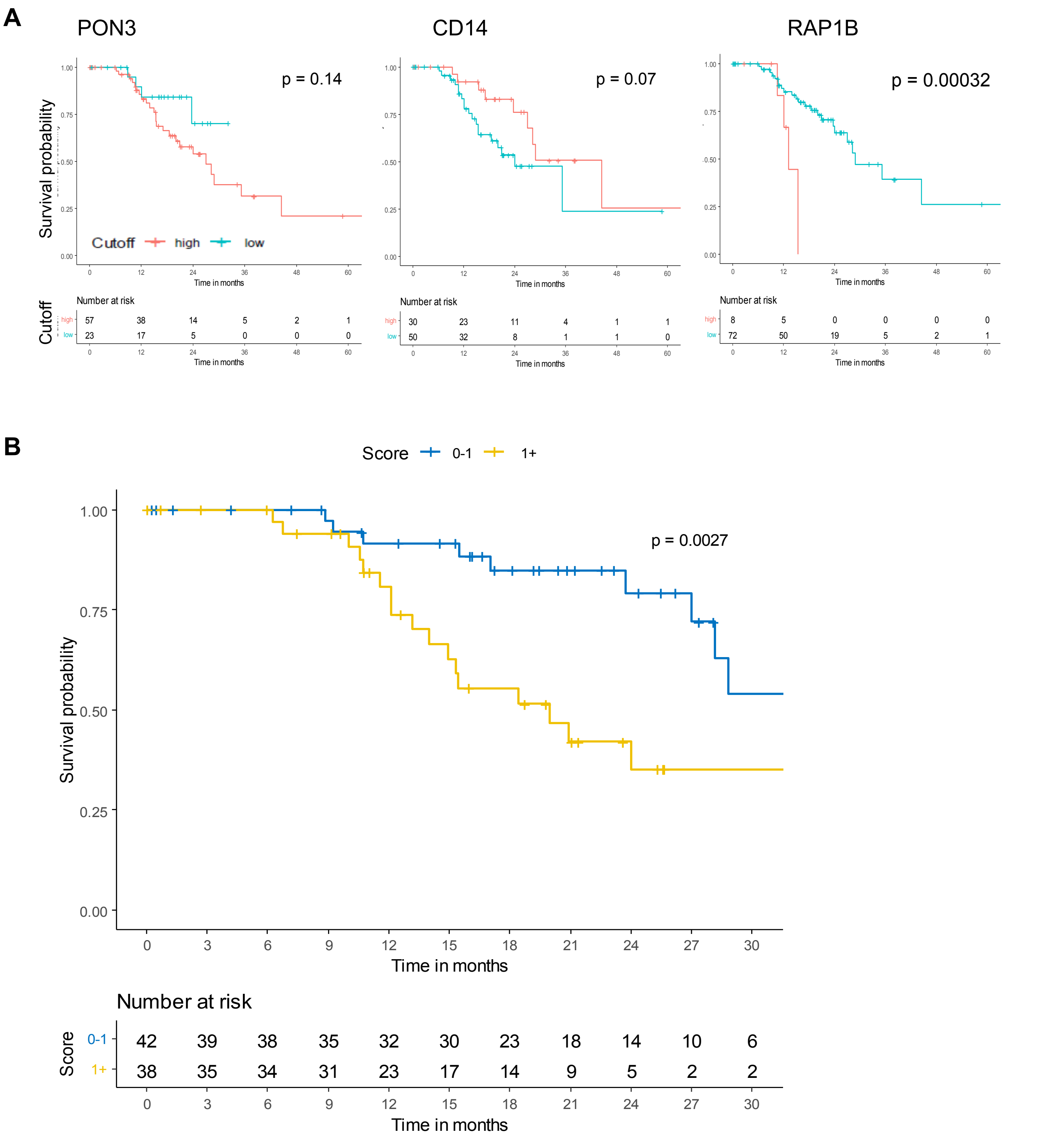

Figure S4. **Association between the 3-gene signature and overall survival of PM patients (TCGA). A.** Survival curves using Kaplan-Meier analysis of overall survival of PM (n=80) patients stratified into high- and low-risk groups based on the cutoff values of each single gene. **B.** Prognostic score built with the 3 genes of A.

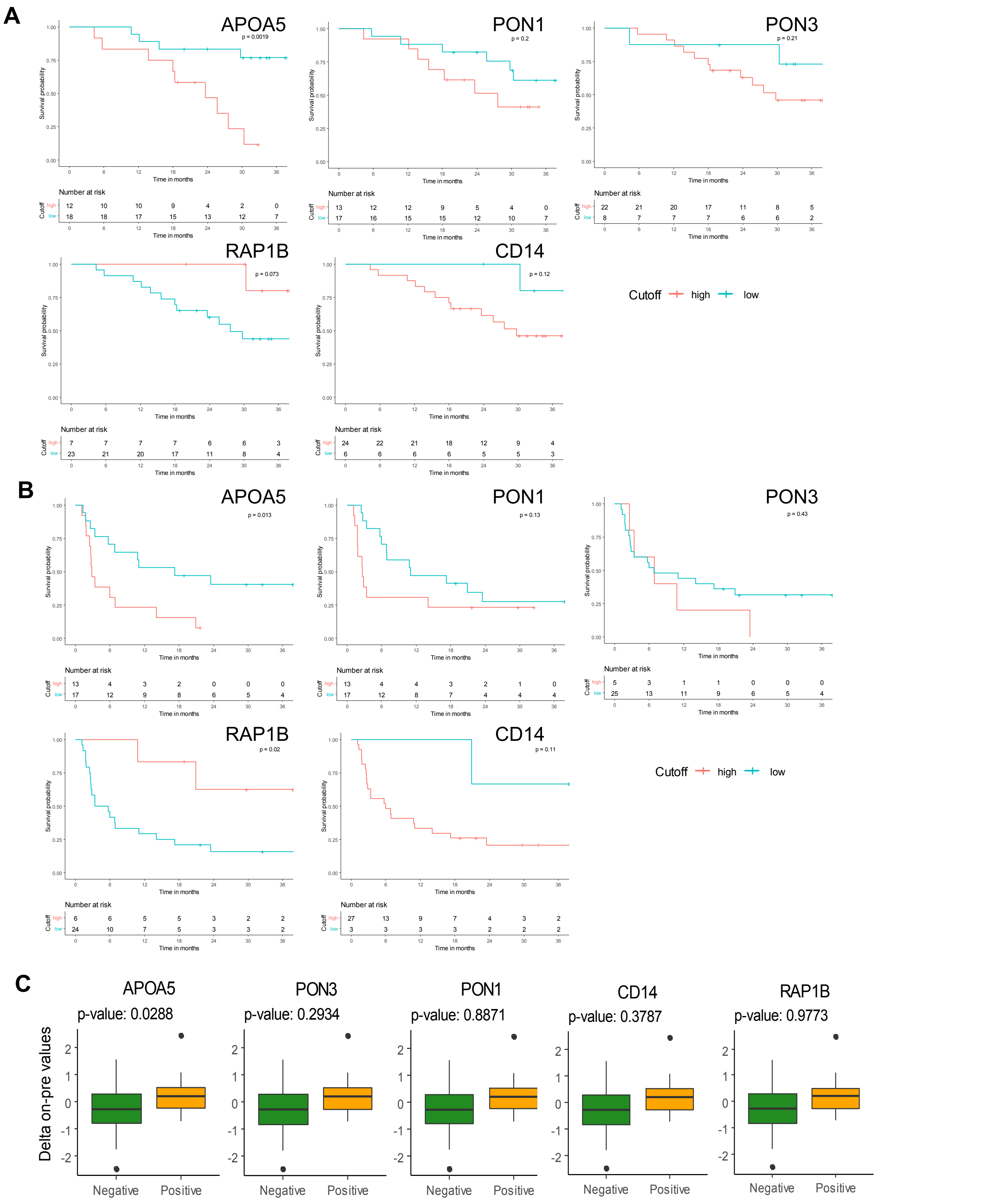

Figure S5. Association between the expression of the five genes by circulating EV, survival and clinical response [55]. Kaplan-Meier curves of OS (**A**) and PFS (**B**) for each gene. **C.** Box plots showing the association between the five genes and clinical response to ICI based on delta values of each gene calculated as *on treatment* values minus *pre* values of gene expression data. Negative indicates non-responders, positive indicates responding patients.
